# Supplementary material for: Therapeutic potential of PRMT1 as a critical survival dependency target in multiple myeloma
Source: BMC Cancer. 2025 Nov 4;25:1704. doi: 10.1186/s12885-025-15104-w (PMC12584455; doi:10.1186/s12885-025-15104-w)
Supplement: Supplementary file 3 — Supplementary Material 3. [file 12885_2025_15104_MOESM3_ESM.docx]

**Supplementary Figure S1**

**
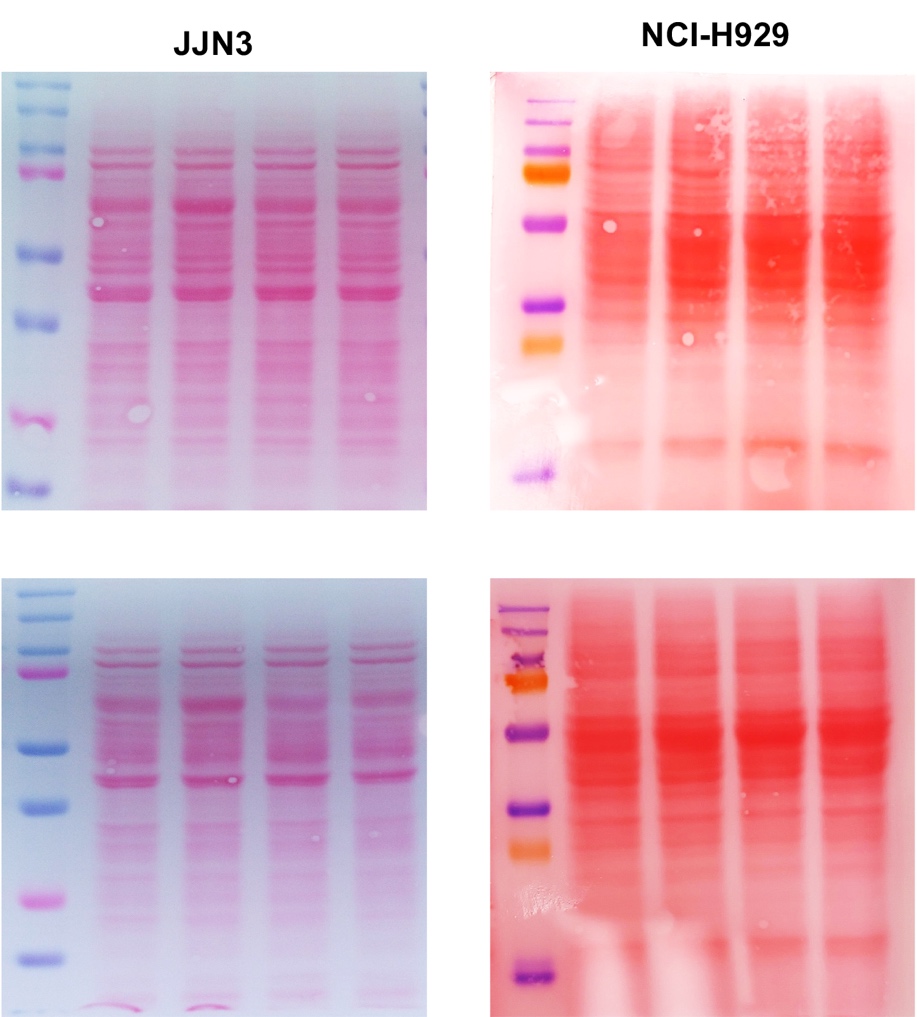
**

**Supplementary Figure S1 | Ponceau staining confirms equal protein loading.** Ponceau staining of membranes from ADMA and MMA western blots in JJN3 and NCI-H929 cells, confirming equal protein loading.

**Supplementary Figure S2**

**
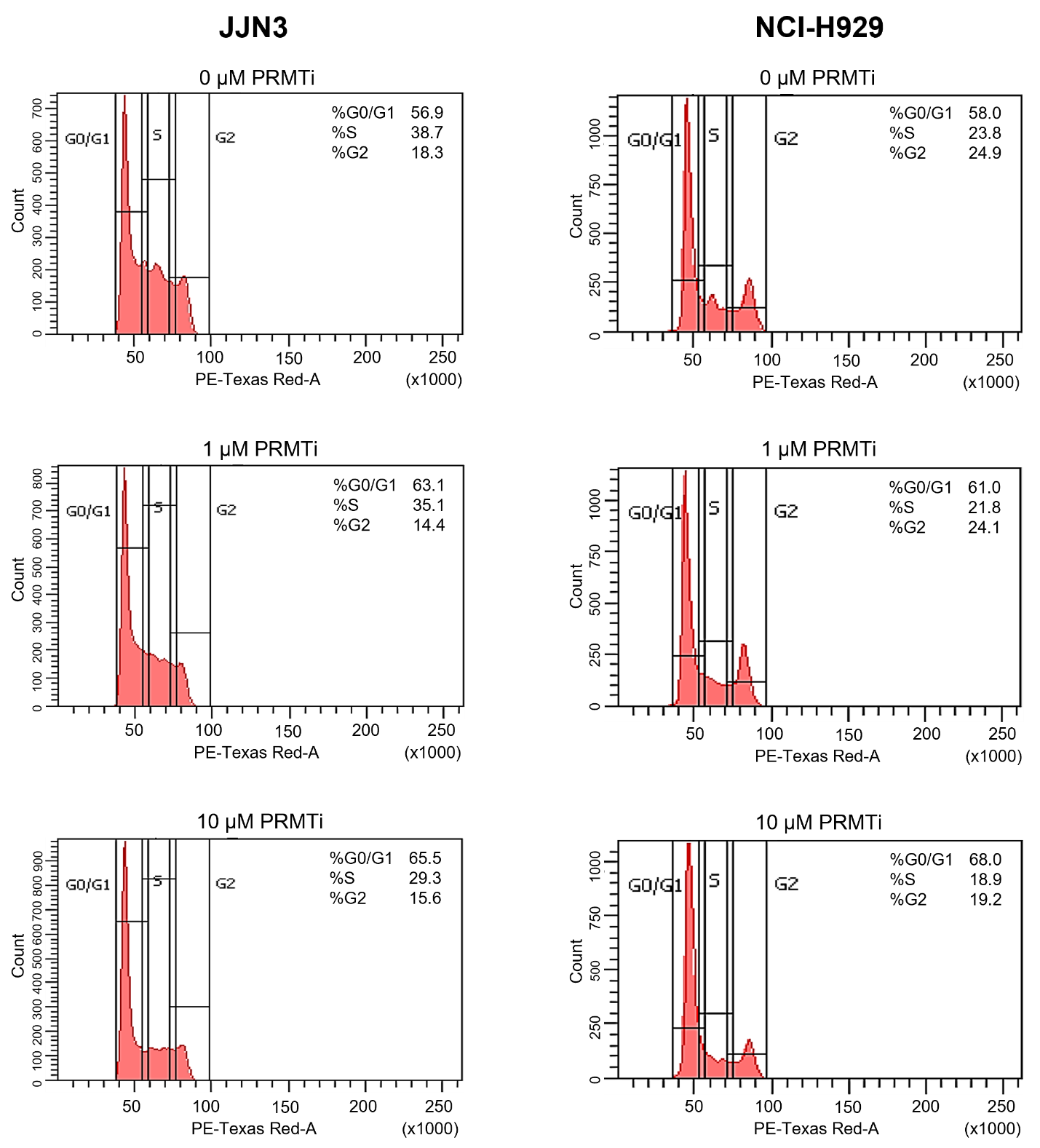
**

**Supplementary Figure S3 | PRMT1 inhibition triggers cell cycle progression defects.** Representative histograms showing DNA content profiles of JJN3 and NCI-H929 cells under the indicated PRMTi concentrations. The percentage of cells in G0/G1, S, and G2/M phases are shown within each panel.

**Supplementary Figure S3**

**
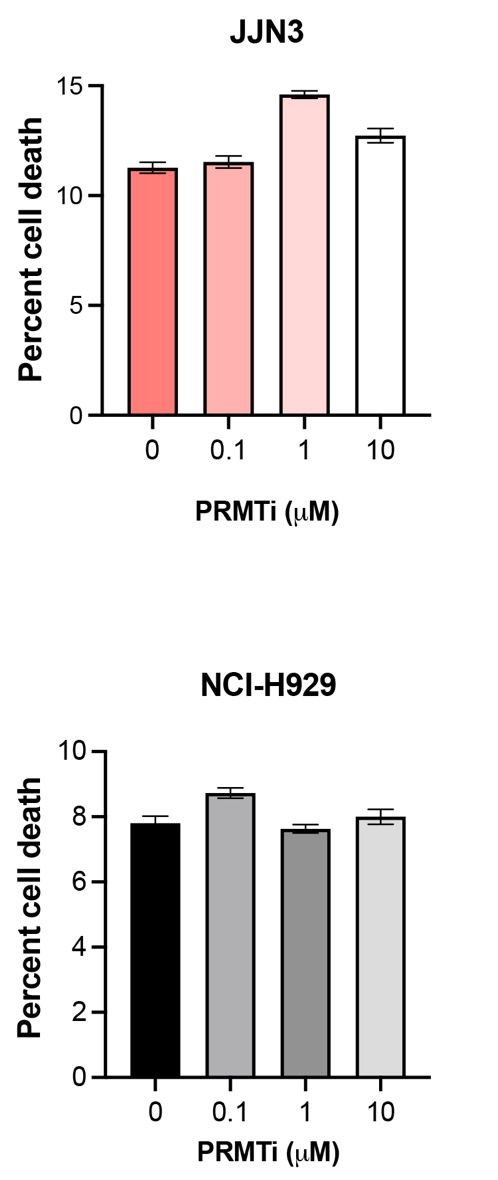
**

**Supplementary Figure S3 | PRMT1 inhibition does not increase apoptotic cell death.** Bar graph showing the percentage of apoptosis and cell death measured by Annexin V-PI staining of JJN3 and NCI-H929 MM cells 72 hours post-PRMTi treatment. Data are presented as the mean ± SEM from three replicates.

**Supplementary Figure S4**

**
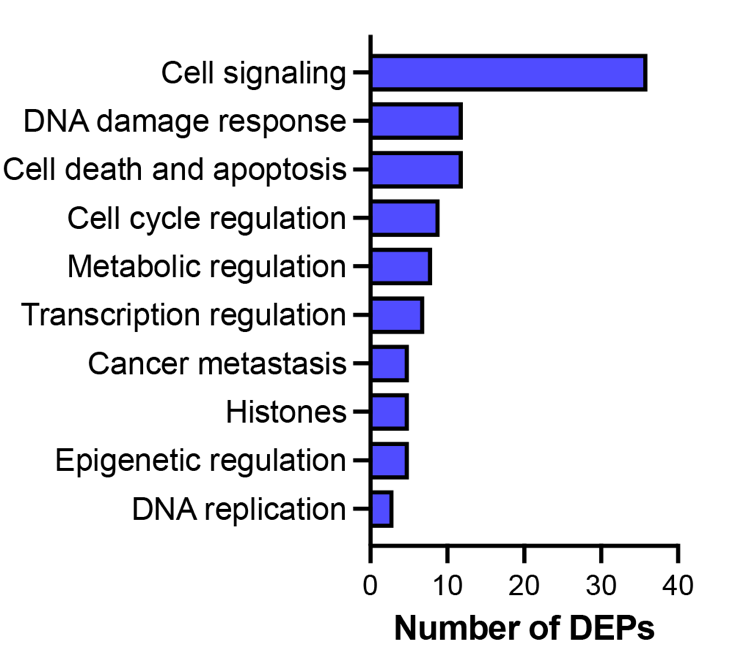
**

**Supplementary Figure S4 | Distribution of 102 DEPs across key molecular functions.** Bar graph showing the distribution of 102 DEPs categorized by their involvement in key molecular functions.
